# Optimization of Electro-Discharge Texturing Parameters for Steel Sheets’ Finishing Rollers

**DOI:** 10.3390/ma13051223

**Published:** 2020-03-09

**Authors:** Emil Evin, Miroslav Tomáš, Jozef Kmec

**Affiliations:** 1 Department of Automotive Production, Faculty of Mechanical Engineering, Technical University of Košice, Mäsiarska 74, 040 01 Košice, Slovakia; miroslav.tomas@tuke.sk; 2 Department of Physics, Mathematics and Technics, Faculty of Humanities and Natural Sciences, Presov University, Ul. 17 novembra 1, 081 16 Prešov, Slovakia; jozef.kmec@unipo.sk

**Keywords:** finishing rollers, controlled texture, statistical methods, mean arithmetic roughness, peak count, reliability indices, ANOVA, design of experiment

## Abstract

Exterior car-body parts are made of steel or aluminum sheets. Their formability and appearance after painting depends not only on the mechanical properties but also on their surface texture. The surface roughness characteristics, the roughness average *Ra* and the peak count *Pc* per centimeter depend on the texture of rolling mill’s finishing rollers, their wear and the degree of removal by the rolling mill. The research was carried out on heat-treated finishing rollers on the surface of which a controlled texture was created by changing the electro-discharge texturing (EDT) parameters. Parameters and the number of electro-discharge texturing experiments were optimized using full four-factor experiment techniques at the upper and lower levels of the parameters in the form of 2^4^. The significance of the impact of individual EDT parameters and their interactions was identified based on the variance results. The ANOVA variance analysis results confirmed that the roughness *Ra* and the peak count *Pc* depend primarily on peak current (*I_p_*), discharge peak voltage (*U_p_*), pulse on time (*P_ont_*) and pulse off time (*P_offt_*). Optimization of the effect of the above parameters on the target roughness *Ra_T,FR_* values and the peak count *Pc_T,FR_* of finishing rollers was performed by the response surface methodology (RSM). Obtained regression models describe relationships between the input parameters of the electro-discharge texturing of finishing rollers and the output characteristics of the *Ra_T,FR_* and the *Pc_T,FR_* texture to a very high degree. The reliability of the electro-discharge texturing process of working rollers was assessed using the process capability index *C_pk_*.

## 1. Introduction

When presented with a choice of similar products sold at a similar price on the market, the customer often makes a choice based on their quality. The concept of quality is very broad and largely depends on the customer’s individual requirements. As the quantity of goods on offer increases, the quality requirements become more and more objective. The quality of products is characterized by a set of measurable features, which allow it to be monitored and controlled. For example, in the case of sheets for the car-body panels, not only are the mechanical properties of the sheets emphasized, the texture of their surfaces also receives due attention to guarantee the formability and appearance of the body parts after painting. The regulated texture of the sheet metal surfaces helps to retain the lubricant on the tool’s contact surfaces during the forming processes and thus contributes to improve their formability and final appearance of body parts after painting [1,2,3].

The sheets for the car-body parts are manufactured in a special mode on the galvanizing line, which includes more thorough strip input control (cleanliness, geometry, surface defects, etc.), annealing speed, galvanizing bath parameters, rolling mill parameters and finishing roller texture parameters (*Ra_FR_* roughness and peak count *Pc_FR_*). During the finishing rolling, the texture of rolling mill’s finishing rollers is transferred to the surface of the steel sheet. Transfer of the texture from rollers to the sheet surface depends largely on removal done by the rolling mill, the texture of finishing rollers and the wear of finishing rollers. The wear of finishing rollers also depends on the texturing technology. When the texture is done by shot blasting technology (SBD) the wear is up to 38%, for electro-discharge technology (EDT) the wear is up to 32%, for hard chromed electro-discharged texture (EDT Hard Chrome) the wear is up to 23% and for Topocrom technology the wear is up to 8% [4,5].

Individual car manufacturers require different target values of sheet metal texture. Hence, Skoda Auto requires for the steel sheets when used for the outside car body parts the arithmetic mean roughness value *Ra* = 1.1–1.6 µm and the peak count *Pc* ≥ 40 cm^−1^; Ford requires the arithmetic mean roughness value *Ra* = 1.1–1.7 µm and the peak count *Pc* ≥ 50 cm^−1^ and Volkswagen requires the arithmetic mean roughness value *Ra* = 1.1–1.6 µm and the peak count *Pc* ≥ 60 cm^−1^ [4,5]. The desired texture values of the sheet metal surfaces cannot be achieved by rolling mills with rollers mechanically blasted with fine-grain shots (SBT—shot blast texturing). Mechanical blasting of working rollers results in a stochastic texture creation, which cannot be controlled. For these reasons, use of stochastic systems of texturing finishing rollers by the method of shot blast texturing has been abandoned in favor of deterministic texturing systems: electric discharge texturing (EDT), laser beam texturing (LBT), electron beam texturing (EBT) or the Topocrom method [4,5,6,7].

In application of electro-discharge texturing, the material is removed by repeated discharges that cause local melting or evaporation of material from the surface of the heat-treated roller [8]. The resulting texture of working rollers depends on the size of craters, or on the amount of material removed *Q_vi_.*

(1) $$Q_{vi}=k\cdot f\cdot r\cdot k_{e}\cdot E_{i}\;({\mathrm{mm}}^{3}\cdot s)$$

where the amount of energy of a single discharge

(2) $$E_{i}=\int_{0}^{t}U_{P}\left(t\right)\cdot I_{P}\left(t\right).dt\;(J)$$

where *k* is the proportionality factor for the anode and cathode; *f* is the discharge frequency (s^−1^); *r* is the electric discharge efficiency (%); *k_e_* is the generator efficiency (%); *E_i_* is the single discharge energy (J); *t* is the length of discharge duration—*P_ont_* (µs); *U_P_* is the discharge voltage (V) and *I_P_* is the peak current (A).

Laser beam texturing (LBM) allows for creation of a regular or pseudo stochastic surface texture with crater overlay and increased wear resistance of the working rollers. Electro-discharge texturing (EDT) can produce a wide range of *Ra_FR_* roughness from 0.5 to 10 μm with a *Pc_FR_* peak count up to 150 cm^−1^, with a uniform microrelief surface, good reproducibility of the surface texture of the finishing roller and its transfer to the sheet surface [7]—Table 1. Likewise, electron beam texturing (EBT) opens a large window into creating surfaces with a wide range of *Ra_FR_* parameters. However, the texture obtained is not the most desirable in terms of tribological conditions on contact surfaces of the die, since worsened sheet formability can be expected as a result.

Topocrom texturing of the working rollers is based on the elimination of chrome hemispherical segments on the surface of the finishing rollers. The number and dimensions of hemispherical segments (*Ra_FR_* roughness and the *Pc_FR_* texture peak count) can be varied to a large extent with a small scatter along the entire length of the finishing roller by changing parameters of electrolytic deposition. Long service life is typical for finishing rollers made by the Topocrom technology [5]. 

EDT, LBT, EBT and Topocrom working roller processes are progressive, but considerably complicated. Setting these process parameters by trial and error techniques is ineffective. By optimizing the input parameters of these processes, it is possible to control the process of texturing the finishing rollers to meet the ever-increasing surface quality requirements of sheets intended for bodywork parts. 

Studies related to the EDM have shown that the process performance can be considerably improved by properly selecting the process material and operating parameters. Since the EDM process has a very complex nature due to the complicated discharge mechanisms and their interactions, parameter optimization appears to be a hot research area [9]. In [10] authors optimized process parameters after die-sinking EDM of tool steel. They suggested mathematical models for the determination of the optimal combination of significant technological parameters in order to minimize microhardness and total HAZ depth variations of tool steel EN X32CrMoV12-28 after die-sinking EDM with a SF-Cu electrode. Optimization of EDM machining parameters of Inconel 600 was presented in [11]. They analytically modeled the energy density, which is being absorbed by the workpiece and the electrode, and experimentally confirmed that the negative polarity leads to a higher material removal rate, higher electrode wear and higher surface roughness. The effect of pulse current and pulse duration in die-sinking EDM on the machining characteristics of Ti-6Al-4V alloy was studied in [12]. Authors used an electrode wear ratio, the material removal rate and the surface roughness to measure the effect of machining and control charts for controlling the process. EDM process parameters were optimized also for the shape-memory alloy NiTi 60 [13] by applying Taguchi‘s method considering an orthogonal array of L27 and using Minitab software. In [14] authors used the Taguchi method when studying the effect of the peak current, pulse on time and feed rate on the material removal rate at the EDM of tool steel H−13. In [15] authors used the design of experiment to investigate surface layers properties including roughness 3D parameters, the thickness of the white layer, heat affected zone, tempered layer and occurring micro cracks at EDM of tool steel. Besides, a new method of the EDM process, such as ultrasonic vibration assisted EDM process was developed and optimized [16].

Design of experiments (DoE) techniques provide powerful tools for optimizing process parameters. The design of experiment plan lies in understanding the effect of different variable factors and their interactions. The design of experiment (DoE) is based on different settings of input parameters and observation of corresponding output response. The aim of the designed experiment is to find the relation between independent variables *x_i_* and dependent variable *y_i_* in mathematical terminology, or the cause and effect relationship. Optimized input parameters obtained by means of design of experiment techniques (DoE) make it possible to take effective measures related to texture control [17,18,19].

## 2. Design of Experiments, Materials and Methods 

At the beginning of the experiment design, input EDT parameters (factors) were defined: peak current *I_p_*, voltage discharge voltage *U_p_*, pulse-on time (pulse length) *P_ont_* and pulse-off time (the length of a technological pause) *P_offt_* and other electro-discharge texturing factors (roller speed, feed rate, shape and surface area of electrodes used, material of electrodes used), which may have an effect on the monitored target values of finishing rollers *Ra_T,FR_* and *Pc_T,FR_* [9,20,21,22].

The draft design of the experiment sequence was based on Volkswagen’s requirements for the texture characteristics of the sheet steel surfaces intended for bodywork auto body parts. We drew on an assumption that the texture of rolling mill’s finishing rollers was transferred to the steel sheet’s surface during cold rolling as a function of removal and wear of the rollers, or it depends on the number of sheets rolled. This means that the final values of surface texture characteristics *Ra_SS_* and *Pc_SS_* of steel sheets can be controlled by controlling the removal made by the rolling mill. The greater the removal, the greater the contouring (transfer) of texture to the sheet surface. For example, a 30–40% improvement in texture transfer was noted upon the change in removal from 0.7% to 1.1%. Transfer of texture from the roller to the sheet surface is limited by the maximum possible removal value. As aforementioned, in rollers prepared by the EDT in combination with chrome plating, a reduction in roughness transmission of *Ra_FR_* by approximately 25% was due to the roller wear. From these assumptions, the mean value of the target roughness of finishing rollers was established to be 1.6 times the upper roughness value required by Volkswagen on the *Ra_UCL_* sheet metal surface (*Ra_UCL,SS_* = 1.6 μm − upper control line for steel sheet roughness). Thus, the assumed mean target roughness value of finishing rollers *Ra_T,CL,FR_* = 1.6 × *Ra_UCL,SS_* ≈ 2.5 μm. The lower limit of the target roughness value of the designed experiment was set at 2/3 of the upper zone of the maximum roughness value of the sheet metal surface required by the automaker Volkswagen [22,23], i.e., *Ra_T,CL,FR_* = *Ra_UCL,SS_* − 1/3*T_SS,Ra_* = 1.53 µm. The upper limit of the target roughness value of the designed experiment was designed to be 3.8 µm (*Ra_UCL,FR_* = 3.8 µm) also taking into account the roughness tolerance of the working rollers *T_FR,Ra_*. As mentioned, the required roughness values of the sheets intended for the body parts of the car bodies range from 1.1 to 1.7 µm or the surface roughness tolerance of sheets intended for body surface parts is *T_SS,Ra_* = ±0.25 µm. For each target roughness value, estimated *Pc_FR_* peak counts were calculated according to Equation (3) [6]:

(3) $$Pc_{FR}=12.9Ra^{2}-102.9Ra+272\;({\mathrm{cm}}^{-1})$$

According to Equation (3), the target roughness value of the finishing rollers *Ra_T,FR_*_1_ = 1.538 μm will correspond to the peak count *Pc_T,FR_*_1*,min*_ = 144 cm^−1^, *Ra_T,FR_*_2_ = 2.5 μm will correspond to the peak count *Pc_T,FR_*_2*,min*_ = 94 cm^−1^ and *Ra_T,CUL_* = 3.8 μm will correspond to the peak count *Pc_T,FR_*_3*,min*_ = 67 cm^−1^. Texturing was performed on the EDT 2100/4500 device in BP250 oil dielectric with 8 copper electrodes at three different levels of electro-discharge texturing input parameters—Table 2.

Sixteen finishing rollers for each level were subjected to observation. Table 3 shows values measured on the monitored surface texture characteristics (responses) of the finishing rollers *Ra_T,FR_* and *Pc_T,FR_* at the individual EDT parameter levels. The average value (AV) of the investigated output characteristic *Ra_T,FRi_*, or *Pc_T,FRi_* was calculated for each data group and so was the standard deviation SD. Finishing roller texture characteristics obtained at three levels of input parameters were evaluated by the Hommel Tester T1000 roughness meter (JENOPTIK Industrial metrology, Villingen-Schwenningen, Germany) along the entire roller length at 9 locations (at three locations at the left edge of the roller, at three locations at the right edge of the roller, and at three locations at the center of the roller) in the respective planes when the roller got turned by 0°, 90°, 180° and 270° according to DIN EN ISO 12085 [24]. Cut-off length was *l_r_* = 0.8 mm and *l_n_* = 4.0 mm as specified by the device producer for expected Ra values up to 4 µm. *Ra* and *Pc* values were measured parallel to the roller axis.

Mean values and standard deviations of the observed texture characteristics were calculated from repeated experiments at individual target value levels of designed experiments. The texture characteristics were investigated based on the proposed full Type 2^4^ Factor Plan, varying the *I_p_, U_p_, P_ont_* and *P_offt_* EDT (factor) settings of input parameters. After starting the process, the input parameters changed only at the upper and lower levels. In its individual lines, the experiment design shows the conditions under which the experiments were carried out in random order—Table 4.

Evaluation of the experimental results was carried out using the statistical software Minitab 16. Conversion of natural to dimensionless variables *a_i_* was done using the coding relationship (4):

(4) $$a_{i}=\pm 1=\frac{x_{ij}-x_{0i}}{\Delta x_{i}}$$

where the baseline *i*-th factor *x_oi_* = (*x_Hi_* + *x_Di_*)/2 and *i*-th factor variation interval ∆*x_i_* = (*x_Hi_* − *x_Di_*)/2 [25,26].

Using statistical methods of the designed experiment in Minitab 16 makes it possible to:

- Mathematically describe the dependence of the texture characteristics of the finishing rollers *Ra_FRi_* and *Pc_FRi_* on the input factors *I_p_, U_P_, P_ont_* and *P_offt_*;
- Find the optimal combination of input factor settings,
- Filter out noise factors or their interaction in carrying out a minimum number of experiments [25,26].

## 3. Analysis of the results obtained 

**Figure 1:**
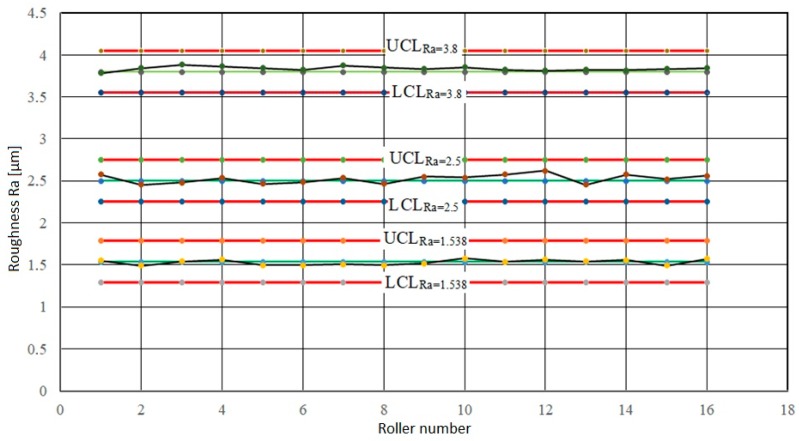
Control charts for target roughness values *Ra_T,Fri_*.

**Figure 2:**
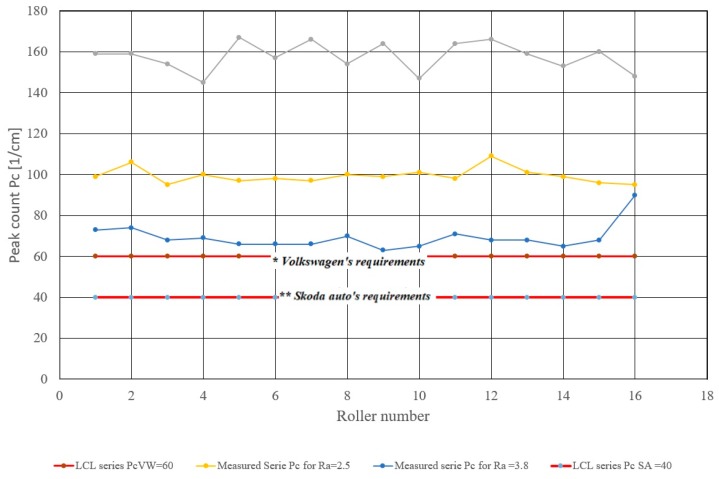
Control charts of the minimum peak count *Pc_min_*.

The calculated mean values of the target roughness values *Ra_T,FR_*_1_ = 1.5358 µm, *Ra_T,FW_*_2_ = 2.5 µm and *Ra_T,TW_*_3_ = 3.8 µm were plotted in control charts—Figure 1 and Figure 2. In control charts, central lines (central line *CL_Ra_*) have been indicated, the values of which equal the target values of the analyzed characteristics *Ra_T,FRi_* and *Pc_T,FRi_* for each range level. Subsequently, control limits, i.e., the lower control limit (*LCL_Ra_*—lower control limit) and the upper control limit (*UCL_Ra_*—upper control limit) were indicated, the values of which were calculated from target values of monitored texture characteristics of working rollers and their tolerances—Figure 2. In establishing the *LCL_Ra_* and *UCL_Ra_* control limits, we assumed that the texture of the finishing rollers is transferred to the surface of the steel sheet during cold rolling as a function of removal and the number of the sheets rolled (wear of the working rollers).

(5) $$T_{SS,Ra}=T_{T,FR,Ra}=\pm 0.25\;\mu m$$

then

(6) $$UCL_{Ra}=Ra_{T,FRi}-T_{SS,Ra}/2$$

(7) $$LCL_{Ra}=Ra_{T,FRi}+T_{SS,Ra}/2$$

and the EDT process capability index:

(8) $$C_{pkUCL}=\frac{UCL-\bar{y}}{3T_{FR}/2}$$

(9) $$C_{pkLCL}=\frac{\bar{y}-LCL}{3T_{FR}/2}$$

where $\bar{y}$ is the mean value of the monitored output characteristics *Ra_T,FRi_* and *Pc_T,Fri_*_,min_ in individual groups [27].

Figure 1 shows that the 1–16 roller roughness values with the *Ra_T,FRi_* roughness values were in the first band (1/3 of the tolerance) of the control boundaries. The calculated *C_pk_* values in all three cases were greater than 1.33. This means that the EDT work procedures applied to each target group were capable of repeatedly achieving the desired target roughness values *Ra_T,FRi_*.

As mentioned in previous research, automakers require different minimum values for the peak count *Pc_SS,min_* per centimeter, ranging between 40 and 60 cm^−1^ in sheets intended for the bodywork parts. If the minimum value of the monitored output characteristic is required, it is indicated in the control charts as *LCL_Pc_* as it is shown in Figure 2:

(10) $$LCL_{Pc}=PC_{SS,min}$$

The position of the central line in control charts was:

(11) $$CL_{Pc,min}=LCL_{Pc}+SD_{Pc,max}$$

where *T_Pc_* is the peak count tolerance *T_n_* = *SD_Pc_,_max_* = 7 cm^−1^ or 3 cm^−1^ as it is shown in Table 3.

Figure 2 shows that the process of roller texturing should be capable of repeatedly creating a texture with target roughness values *Ra_T,FR_*_1_ = 1.538 and *Ra_T,FR_*_2_ = 2.5 µm with *Pc_T,VW,min_* = 60 cm^−1^ as required by the Volkswagen. Since the measured peak count values were above the lower *LCL_Pc_* = *Pc_VW_* limit and the process capability index was greater than 1.33—see Table 3. Peak counts for target roughness values *Ra_T,FR_*_3_ = 3.8 µm were below the lower limit of the required minimum peak count, and *C_pk,Pc_* capability indices were less than 1.33—see Figure 2 and Table 3. This means that the process of texturing the finishing rollers with the target value *Ra_T,FR_*_3_ = 3.8 µm was not capable of repeatedly achieving the texture with the peak count greater than 60 cm^−1^. When setting the EDT process parameters of the finishing rollers listed in Table 2, it can be assumed that for the required values of *Ra_T,FR_* = 2.5 µm and *Ra_T,FR_* = 3.8 µm, requirements of both Ford and Skoda could also be met (*Pc_T,SA,min_* = 40 cm^−1^). For target roughness values ranging from 1.538 to 3.5 µm and a minimum peak count greater than 40, the process capability index *C_pk,Pc_* > 1.33—see Table 3.

### 3.1. Testing the Significance by a t-Test

**Figure 3:**
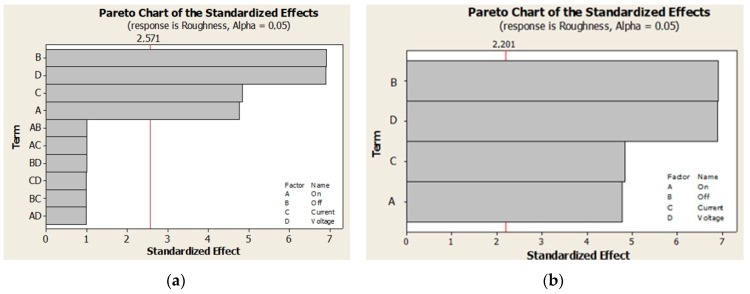
Effect of individual factors on Ra roughness: (**a**) with noise and (**b**) without noise.

**Figure 4:**
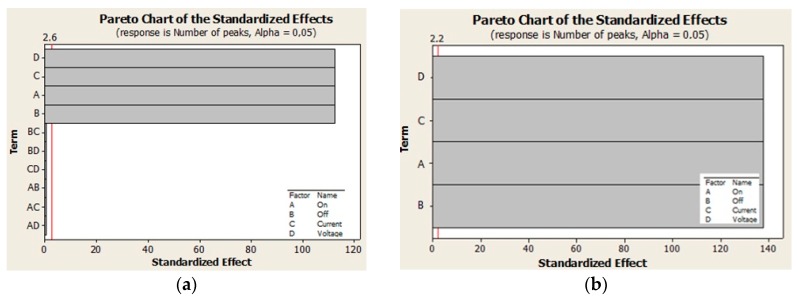
Effect of individual factors on Pc peak count: (**a**) with noise and (**b**) without noise.

Based on the above results, further research focused on finding an input parameter setting that would make it possible to obtain the *Ra_FRi_* values ranging between 1.538 and 3.5 µm while maintaining the peak count of *Pc_F,WV,min_* > 60 cm^−1^ or *Pc_F,SA,min_* > 40 cm^−1^. In order to achieve this goal, the results were analyzed using a complete factor experiment 2^4^, with the application of mathematical-statistical methods in the environment of Minitab 16—see Table 5, Table 6, Table 7 and Table 8. The *F*-test and the *t*-test, respectively were used to test the significance of the influence of parameters applied to the EDT process levels on the roughness *Ra_T,FRi_* and the peak count of *Pc_T,FR,min_*. The results of the ANOVA analysis done in Minitab 16 software at a significance level of 95% using the *t*-test and the *F*-test were processed graphically and tabularly—Table 5, Table 6, Table 7 and Table 8 and Figure 3 and Figure 4.

The hypothesis tested is the effect of the factors tested or that of their interactions to the surface texture parameters is significant. The statistical significance of individual factors and their interaction was expressed using the *p*-value. If *α* ≥ *p*, the null hypothesis *H*_0_ is rejected and the alternative hypothesis *H*_1_ is accepted. This means that the effect of factors tested or that of their interactions is significant for a given significance level α. Conversely, if *α* < *p*, the null hypothesis *H*_0_ must be accepted. This means that the effect of individual factors or their interactions is insignificant [25,26].

The signal-to-noise (S/N) ratio method was used to optimize the selected response variables. Based on the selected response characteristic, a larger-the-better S/N ratio has been used in this research work [27,28]. It follows from the analysis presented in Table 5 and Table 6 that the *p*-value for individual factors was less than 0.05, therefore the alternative hypothesis *H*_1_ was accepted. The average roughness values *Ra_T,FRi_* at the set levels of factors *I_P_, U_P_, P_ont_* and *P_offt_* were not equal to each other, therefore, it could be stated that their influence on the significance level α = 0.05 was statistically significant. In case of mutual interactions of the monitored factors, the null hypothesis was not rejected because the *p*-values of the test criterion were greater than 0.05, their effect on the resulting roughness at the significance level α = 0.05 was statistically insignificant. 

On the basis of the calculated regression coefficients shown in Table 5, it is possible to write a model regression equation predicting the *Ra_FRi_* roughness with the noise taken into account in the following form:

(12) $$\begin{array}{c} Ra_{FRi}=2.58+0.222P_{ont}+0.321P_{offt}+0.225I_{P}+0.32U_{P}+0.047P_{ont}P_{offt}-0.047P_{ont}I_{P}\\ +0.046P_{ont}U_{P}+0.046P_{offt}I_{P}-0.047P_{offt}U_{P}+0.046I_{P}U_{P} \end{array}$$

and after neglecting the noise, the model regression equation can be written in a simpler form:

(13) $$Ra_{FRi}=0.906+0.0177P_{ont}+0.0201P_{offt}+0.03I_{P}+0.0366U_{p}$$

The overall informative level of the prediction Model (12) and Model (13) was given by the multiple determination coefficient *R-Sq (adj)* = 90.27% and the correlation coefficient *R* = 0.95. Based on the multiple determination coefficient *R-Sq (adj)* and the correlation coefficient, it can be stated that Model (12) and Model (13) could predict the roller roughness with a probability of 95%.

Similarly as mentioned for the average roughness values *Ra_T,FRi_*, the model predicting the peak count based on calculated values of regression coefficients shown in Table 6 can be written in the form:

(14) $$\begin{array}{c} Pc_{FR}=112.7-11.31P_{ont}-11.31P_{offt}-11.31I_{P}-11.31U_{P}-0.06P_{ont}P_{offt}-0.06P_{ont}\\ -0.06P_{ont}U_{P}-0.06P_{offt}I_{P}-0.06P_{offt}-0.06I_{P}U_{P} \end{array}$$

and after neglecting the noise, the model regression equation can be written in a simpler form:

(15) $$PC_{FR}=181.5-0.905P_{ont}-0.707P_{offt}-1.508I_{P}-1.293U_{p}$$

The value of the multiple regression coefficient *R-Sq (adj)* model for the *Pc_FR_* peak count prediction was 100%, and similarly, the correlation coefficient R = 1. Thus, it can be stated that Models (14) and (15) could predict the peak count with 100% probability.

From the analysis of deviations of factors listed in Table 5 and Figure 3 it follows that regardless of the fact whether noise is or is not taken into account, the greatest influence on the *Ra_FR_* roughness is that of the *P_offt_* factor and the smallest is that of the *P_ont_* factor, while their interactions are insignificant. From the analysis of deviations of factors listed in Table 6 and Figure 4 follows that individual factors *P_ont_, P_offt_, I_P_* and *U_P_* have the same effect on the *Pc_FR_* peak count, while their mutual interactions are insignificant.

### 3.2. Testing the Significance by the F-Test

The results obtained from the designed experiment were also tested using the *F*-test, which is based on the significance of the difference between two variations. The decomposition of total variability into its individual parts makes it possible to find out the main source of variability and factors that show a significant effect on the resulting values of *Ra_FR_* roughness and the *Pc_FR_* peak count. The result of the test is a comparison of the critical value of *Fα; a−1; N-a* with the value of the test criterion *F*. Similarly to the *t*-test, the *F*-test makes it easier and quicker to establish the significance of individual factors using the *p*-value. Table 9 and Table 10 show that the *p*-value for each factor was less than 0.05, and Table 11 shows estimated coefficients for roughness and for the number of peaks. This means that the effect of individual factors (*P_ont_, P_oft_, I_P_* and *U_P_*) on the average roughness values of *Ra_FRi_* was statistically significant. As shown in Table 9 and Table 10, in cases of a mutual interaction of the monitored factors, the null hypothesis could not be rejected, because the *p*-values of the test criterion were greater than 0.05—the mutual interactions of the factors were insignificant.

On the basis of the calculated regression coefficients shown in Table 11, it is possible to write a model regression equation predicting the *Ra_FR_* roughness with the noise taken into account in the following form:

(16) $$\begin{array}{l} Ra_{FRi}=1.121+0.0114P_{ont}+0.017P_{offt}+0.018I_{P}+0.029U_{P}+0.00023P_{ont}P_{offt}\\ \;\;\;-0.000496P_{ont}I_{P}+0.00042P_{ont}U_{P}+0.00039P_{offt}I_{P}-0.00033P_{offt}U_{P}\\ \;\;\;+0.00071I_{P}U_{P} \end{array}$$

On the basis of the calculated regression coefficients shown in Table 11, it is possible to write a model regression equation predicting the *Pc_FRi_* roughness with the noise taken into account in the following form:

(17) $$\begin{array}{c} Pc_{FR}=181-0.881P_{ont}-0.689P_{offt}-1.47I_{P}-1.263U_{P}-0.000312P_{ont}P_{offt}-0.00067P_{ont}I_{P}\\ -0.000571P_{ont}U_{P}-0.000521P_{offt}I_{P}-0.000446P_{offt}U_{P}-0.00095I_{P}U_{P} \end{array}$$

**Figure 5:**
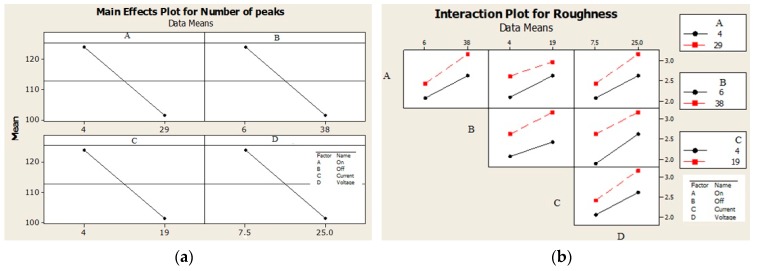
Plot for *Ra_FR_* roughness: (**a**) the main effect and (**b**) interactions.

**Figure 6:**
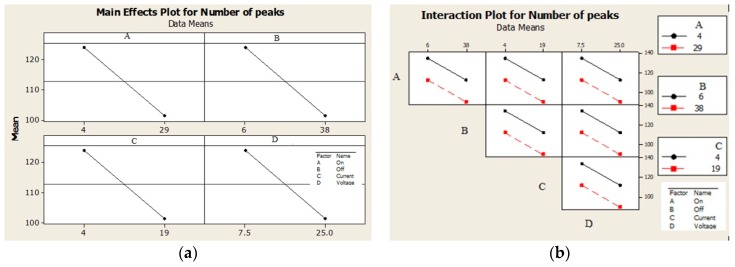
Plot for *Pc_FR_* peak count: (**a**) the main effect and (**b**) interactions.

Figure 5 shows that with increasing values of investigated factors (*I_P_, P_ont_, P_offt_* and *U_P_*) between the lower and upper levels, the *Ra_FRi_* roughness increased. In the case of an increase in the *Ra_FRi_* roughness, the increase was smaller at the lower level of the factors than at the upper level of the factors investigated—Figure 5b. This means that by increasing the EDT parameters at the upper level, a significant improvement in the spark discharge effect was achieved. Depending on the peak count of the individual investigated factors (Figure 6) it follows that with increasing values of investigated factors in the range between the lower and the upper level, the opposite effect was achieved, i.e., with increasing values of the investigated EDT factors, there was a decrease in the number of *Pc_FRi_* peak count. Greater effect can be expected when changing the parameters of the EDT process in the lower level area of individual factor parameters than in the area of the upper level—Figure 6b.

The *F*-test results confirmed that the parameters applied to the roller texturing process should be capable of repeatedly creating the texture with *Ra_T,FR_*_1_ = 1.538 µm as well as the texture with *Ra_T,FR_*_2_ = 2.5 µm, both fulfilling *Pc_T,FR_*_1*,min*_ > 60 cm^−1^, as required by the automaker Volkswagen. Since the measured peak count values were above the lower *LCL_Pclimit_* and the process capability index was greater than 1.33. Table 3 and Figure 2 show that the process of texturing the working rollers with the target value *Ra_T,FR3_* = 3.8 µm was not capable of repeatedly achieving a texture with a peak count *Pc_T,FR_*_1*,min*_ > 60 cm^−1^, because the *C_pkPc_* capability indices were less than 1.33.

When setting the EDT process parameters listed in Table 2, it can be assumed that the texture with *Ra_T,FRi_* will be repeatedly achieved, ranging between 1.538 and 3.8 µm and, at the same time, the peak count *Pc_T,FR_*_2*,min*_ > 40 cm^−1^, as required by Skoda. At the target roughness values ranging from 1.538 to 3.5 µm, the minimum peak count was greater than 40 and the capability index *C_pkPC_* > 1.33—Table 4.

The C-E (Cause and Effects) analysis of errors on the finishing rollers after EDT showed that the rollers are most often damaged by burns, forming strips along the roller circumference and its length. The roller gets burns due to setting of a short technological pause or low speed, which does not eliminate impurities from the dielectric fluid and reforms the arc between the electrode and the finishing roller. This undesirable effect can be avoided by prolonging the technological pause at an interval at which the electric discharge channel regenerates in the spark gap. If this does not correct the error, the roller speed must be increased. The risk of other errors can be eliminated by regular maintenance.

## 4. Optimization of the EDT Factors’ Effect on Surface Texture Characteristics

Response surface methodology (RSM) was used to optimize the EDT factors’ effect on surface texture characteristics. RSM is a collection of mathematical and statistical techniques useful for analyzing problems in which several independent variables influence a dependent variable or response, and the goal is to optimize this response. In many experimental conditions, it is possible to represent independent factors in the quantitative form [29].

**Figure 7:**
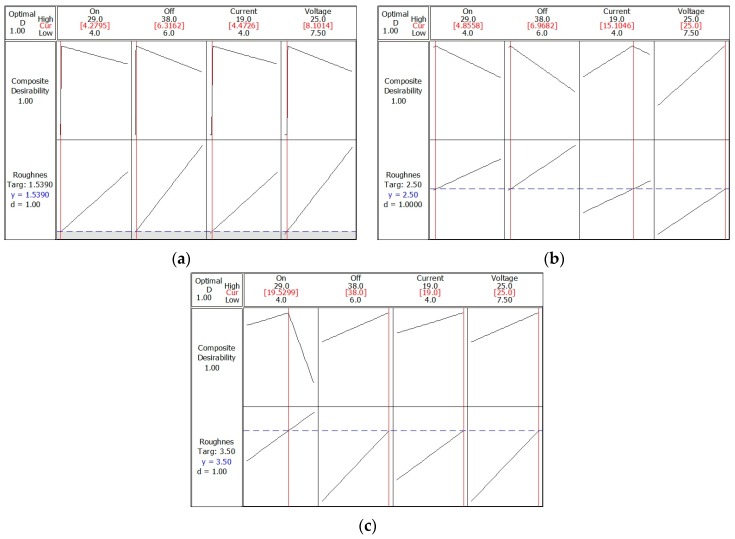
Optimization of EDT input parameters to achieve the roughness target value: (**a**) *Ra_T,FR_*_1_ = 1.538 μm; (**b**) *Ra_T,FR_*_2_ = 2.5 μm and (**c**) *Ra_T,FR_*_3_ = 3.5 μm.

Optimal values of EDT input parameters to achieve the target *Ra_T,FRi_* roughness values ranging from 1.538 to 3.8 μm and a minimum peak count *Pc_T,FR_*_1*,min*_ > 60 cm^−1^ (Volkswagen requirement) or minimum peak count *Pc_T,FR_*_2*,min*_ > 40 cm^−1^ (Skoda requirement) were established by the response surface methodology (RSM). These two output characteristics *Ra_T,FRi_* and *Pc_T,FRi,min_* of the surface of finishing rollers show a contradictory tendency. During optimization, priority was given to achieving the target values of *Ra_T,FR_*_1_ = 1.538 μm, *Ra_T,FR_*_2_ = 2.5 μm and *Ra_T,FR3_* = 3.5 μm. The target roughness values of *Ra_T,FRi_*, their lower and upper values, their weight and importance were defined at the input and optimized values of the input parameters (*P_ont_, P_offt_, I_P_* and *U_P_*) were calculated based on these input data. The results of the response optimization for the desired roughness target values *Ra_T,FRi_* are shown in Table 12, Table 13 and Table 14 and Figure 7. Based on the optimized values of the *P_ont_, P_offt_, I_P_* and *U_P_* parameters, the values of the peak count were calculated using the regression models (14) and (17)—Table 12, Table 13 and Table 14.

Figure 7a shows that with the combination of optimized EDT parameters (*P_ont_* = 4.29 μs, *P_offt_* = 6.32, *I_P_* = 4.47 A and *U_P_* = 8.1 V), required target roughness values *Ra_T,FR_*_1_ = 1.539 μm were achieved. The total composite desirability when using RSM for predicted responses or the roughness target was at level 1. Thus, the response (*Ra_T,FR_*_1_) has reached an ideal state—it is within an acceptable interval. The minimum peak count *Pc_T,FR1_* = 156 cm^−1^ was established by calculation according to the regression Models (14) and (17), at which a coefficient of determination *R-Sq (adj)* of 99.97% and a coefficient of correlation *R* = 1 were recorded.

Figure 7b shows that with the combination of the optimized EDT parameters (*P_ont_* = 4.86 μs, *P_offt_* = 6.97 μs, *I_P_* = 15 A and *U_P_* = 25 V), required target roughness values *Ra_T,FR_*_2_ = 2.5 μm were achieved. The total composite desirability when using RSM for predicted responses or the roughness target was at level 1. This means that the response (*Ra_T,FR_*_2_) has reached an ideal state. The minimum peak count *Pc_T,FR_*_2_ = 118 cm^−1^ was established by calculation according to the regression Model (14) and (17), at which a coefficient of determination *R-Sq (adj)* of 99.97% and a coefficient of correlation *R* = 1 were recorded.

Figure 7c shows that with the combination of the optimized EDT parameters (*P_ont_* = 19.5 μs, *P_oft_* = 38 μs, *I_P_* = 19 A and *U_P_* =25 V), required target roughness values *Ra_T,FR_*_3_ = 3.5 μm were achieved. The total composite desirability when using RSM for predicted responses or the roughness target was at level 1. This means that the *Ra_T,FR_*_3_ response had reached an ideal state. The minimum peak count *Pc_T,FR_*_3_ = 78 cm^−1^ was established by calculation according to the regression Model (14) and (17), at which a coefficient of determination *R-Sq (adj)* of 99.97% and a coefficient of correlation *R* = 1 were recorded.

Response surface optimization results confirmed that the parameters applied to the roller texturing process should be capable of repeatedly creating a texture with *Ra_T,FR_*_1_ = 1.538 µm, *Ra_T,FR_*_2_ = 2.5 µm and *Ra_T,FR_*_3_ = 3.5 µm with *Pc_T,FR,min_* > 60 cm^−1^. When setting the EDT process parameters listed in Table 2, it can be assumed that the texture with *Ra_T,FRi_* will be repeatedly achieved, ranging between 1.538 and 3.5 µm and, at the same time, the peak count *Pc_T,FRi,min_* > 40 cm^−1^, as required by Skoda. At the target roughness values ranging from 1.538 to 3.5 µm, the minimum number of peaks was greater than 40—Table 3.

Optimized parameters of EDT factors were verified to reach the target value of the finishing rollers surface roughness *Ra* = 2.5 µm. After applying these parameters (Table 13) the roughness values of the finishing rollers were 2.4 ± 0.06 µm.

## 5. Conclusions

The main aim of this paper was to obtain optimized electro-discharge texturing parameters of rolling mill finishing rollers for surface texture finish values ranging from *Ra_T,FR_* = 1.539 to *Ra_T,FR_* = 3.5 μm, while at the same time, the minimum peak count should be greater than 60 according to Volkswagen and larger than 40 cm^−1^ according to the requirements of Skoda carmakers. The research involved a sample of 48 pieces of finishing rollers (i.e., 16 for each level of parameters), the surface of which was subjected to texturing by the EDT 2100/4500 device in a BP250 oil dielectric with eight copper electrodes at varying levels of input parameters of electro-discharge texturing (EDT), creating textures with the target roughness values *Ra_T,FR_*_1_ = 1.538 μm, *Ra_T,FR_*_2_ = 2.5 μm and *Ra_T,FR_*_3_ = 3.8 μm and the peak count *Pc_FR_*_1_ = 158 cm^−1^, *Pc_FR_*_2_ = 99 cm^−1^ and *Pc_FR_*_3_ = 68 cm^−1^. With the use of control charts and process capability indices *C_pk_*, rollers were selected on which the *t*-test and the *F*-test analyses were subsequently performed. The results of the *t*-test and the *F*-test show that regression models obtained describe the relationships between the EDT input parameters and the *Ra_FR_* and *Pc_FR_* finishing rollers texture characteristics with a very high probability. Significance tests show that at the significance level α = 0.05, a significant effect of the EDT input parameters was noted: the duration of the technological pause *P_ont_*, voltage *U_p_*, current *I_p_* and the length of the technological pause on the texture characteristics *Ra_FR_* and *Pc_FR_*. Influence of mutual interactions of individual input factors on the roughness *Ra_FR_* and the peak count *Pc_FR_* at significance level *α* = 0.05 was not recorded. The Pareto chart shows that the greatest influence on the roller roughness *Ra_FR_* was that of duration of the technological pause and the lowest was that of the current *I_p_*. In terms of the peak count and *Pc_FR_*, the effect of individual factors was at the same level. The resulting roughness of the finishing rollers or the amount of material removed depended on the energy of the electric discharge. With very small changes in *P_ont_* from 4.29 to 4.86 µs, in *P_offt_* from 6.32 to 6.97 µs but larger current changes *I_p_* from 4.47 to 15.1 A and voltage *U_p_* from 8.1 to 25 V there was a change in Δ*Ra_FR_* by 0.961 µm (from 1.539 to 2.5 µm) and Δ*Pc_FR_* by 38 cm^−1^ (from 156 to 118 cm^−1^). At the same voltage *U_p_* = 25 V and current change *I_p_* from 15.1 to 19 A but with major changes in *P_ont_* from 4.86 to 19.53 µs, *P_offt_* from 6.97 to 38 µs, a change in roughness Δ*Ra_FR_* was also achieved by about 1 µm (from *Ra_FR_* 2.5 to 3.5 µm) and a change in *Pc_FR_* by 40 peaks per cm (from 118 to 78 cm^−1^). Thus, based on the results obtained, it could be concluded that a greater effect was achieved by setting the input parameters of EDT at their lower level than at their upper level.

Optimal values of input parameters *I_P_, U_P_, P_ont_* and *P_offt_* were established by the RSM method. Based on the verification of RSM results, it can be stated that the optimal (ideal) values of the target roughness characteristics have been achieved: *Ra_T,FR_* = 1.539 µm and, at the same time, the minimum values *Pc_T,FR,min_* = 156 cm^−1^ when setting the current *I_P_* = 4.47 A, *U_P_* = 8.1 V, *P_ont_* = 4.29 µs, *P_offt_* = 6.32 µs; *Ra_T,FR_* = 2.5 µm and, at the same time, the minimum values *Pc_T,FR,min_* = 118 cm^−1^ when setting the current *I_P_* = 15.1 A, *U_P_* = 25, *P_ont_* = 4.86 µs and *P_offt_* = 6.97 µs; *Ra_T,FR_* = 3.5 µm and, at the same time, the minimum values *Pc_T,FR,min_* = 78 cm^−1^ when setting the current *I_P_* = 19 A, *U_P_* = 25 V, *P_ont_* = 19.53 µs and *P_offt_* = 38 µs.

The results obtained further show that with increasing roughness *Ra_FR_* a decrease in the *Pc_FR_* peak count was observed as was a good correlation between these roller texture characteristics. The obtained set of input parameters can be used to optimize other output characteristics of the EDT process such as cost and can also form the basis for designing adaptive process control strategies for EDT process of finishing rollers.

## Figures and Tables

**Table 1:** Comparison of selected parameters for texturing methods.

	Texturing Method				
Parameter	SBT	EDT	LBT	EBT	Topocrom
Irregularities	Triangular Trapezoidal	Craters	Craters with solid collar	Craters with solid collar	Spherical segments
Topography	Random	Random	Random or determinate	Random or determinate	Random
*Ra* (µm)	1.5–6	0.5–10	0.8–10	0.5–20	0.5–20
*Pc* (cm^−1^)	< 70	50–150	50–100	50–150	50–200

**Table 2:** Input parameters of the electro-discharge texturing process.

Control Factors	Symbol	Unit	Level 1	Level 2	Level 3
Peak current	*I_p_*	A	4	8	19
Pulse-on time	*P_ont_*	µs	4	14	29
Pulse-off time	*P_offt_*	µs	6	16	38
Lower value of discharge voltage	*U_IL_*	V	5	10	15
Upper value of discharge voltage	*U_IH_*	V	8	20	35
Average value of discharge voltage (*U_IL_* + U*_IH_*)/2	*U_p_*	V	6.5	15	25
Electrode diameter	*D*	mm	8	8	8
Target *Ra* value of the finishing rollers	*Ra_T,Fri_*	µm	1.5	2.5	3.8
Target *Pc* value of the finishing rollers	*Pc_T,FRi_*	cm^−1^	140	92	62

**Table 3:** Measured target roughness *Ra_T,FRi_* and peak count values *Pc_T,FRi_* at individual EDM parameter level settings.

Number	Level 1		Level 2		Level 3	
	*Ra_T,FR_*_1_ (μm)	*Pc_T,FR_*_1_ (cm^−1^)	*Ra_T,FR_*_2_ (μm)	*Pc_T,FR_*_2_ (cm^−1^)	*Ra_T,FR_*_3_ (μm)	*Pc_T,FR_*_3_ (cm^−1^)
1.	1.554	159	2.577	99	3.781	73
2.	1.493	159	2.453	106	3.844	74
3.	1.545	154	2.482	95	3.886	68
4.	1.563	145	2.537	100	3.866	69
5.	1.495	167	2.465	97	3.842	66
6.	1.498	157	2.484	98	3.823	66
7.	1.507	166	2.537	97	3.874	66
8.	1.495	154	2.465	100	3.847	70
9.	1.516	164	2.553	99	3.831	63
10.	1.578	147	2.541	101	3.852	65
11.	1.538	164	2.575	98	3.822	71
12.	1.564	166	2.623	109	3.813	68
13.	1.543	159	2.456	101	3.825	68
14.	1.557	153	2.577	99	3.816	65
15.	1.494	160	2.523	96	3.831	68
16.	1.573	148	2.563	95	3.842	90
*AV*	1.532	158	2.481	99	3.837	68
*SD*	±0.03	±7	±0.05	±3	±0.03	±3
*CL*	1.538	122	2.5	122	3.8	122
*UCL*	1.788	-	2.25	-	4.05	-
*LCL*	1.288	60*	2.75	60*	3.55	60*
*C_pk,UCL_*	2.84	-	2.99	-	2.37	-
*C_pk,LCL_*	2.71	4.67	2.57	4.33	3.19	0.89
*CL*	1.538	47	2.5	47	3.8	47
*UCL*	1.788	-	2.25	-	4.05	-
*LCL*	1.288	40**	2.75	40**	3.55	40**
*C_pk,UCL_*	2.84	-	2.99	-	2.37	-
*C_pk,LCL_*	2.71	5.62	2.57	6.56	3.19	3.11

* Volkswagen’s requirements; ** Skoda Auto’s requirements.

**Table 4:** Setting the upper and lower levels of the electro-discharge texture parameters and the full experiment design response 2^4^.

Run Order	Center Pt	Blocks	Analyzed Factors				Responses	
			*P_ont_* (µs)	*P_offt_* (µs)	*I_P_* (A)	*U_P_* (V)	Roughness *Ra_T,FR_* (µm)	Number of Peaks *Pc_T,FR_* (cm^−1^)
1	1	1	29	6	19	6.5	2.750	73
2	1	1	29	38	4	25	2.970	73
3	1	1	4	38	4	25	2.433	95
4	1	1	29	38	19	25	3.820	71
5	1	1	4	6	19	25	3.096	73
6	1	1	4	38	19	25	3.096	73
7	1	1	4	6	19	6.5	2.214	96
8	1	1	4	38	4	6.5	1.551	118
9	1	1	4	6	4	25	2.433	95
10	1	1	4	38	19	6.5	2.214	96
11	1	1	29	6	4	6.5	2.087	96
12	1	1	29	6	19	25	3.632	50
13	1	1	29	38	4	6.5	2.087	96
14	1	1	4	6	4	6.5	1.551	158
15	1	1	29	6	4	25	2.970	73
16	1	1	29	38	19	6.5	2.750	73

**Table 5:** Estimated effects and coefficients for roughness (coded units).

Term	Effect	Coef	SE Coef	*T*	*p*-Value
*Constant*	-	2.57975	0.04645	55.54	0.000
*P_ont_*	0.44350	0.22175	0.04645	4.77	0.005
*P_offt_*	0.64225	0.32112	0.04645	6.91	0.001
*I_p_*	0.45000	0.22500	0.04645	4.84	0.005
*U_p_*	0.64075	0.32038	0.04645	6.90	0.001
*P_ont_*·*P_offt_*	0.09325	0.04663	0.04645	1.00	0.363
*P_ont_*·*I_P_*	−0.09300	−0.04650	0.04645	−1.00	0.364
*P_ont_*·*U_P_*	0.09275	0.04637	0.04645	1.00	0.363
*P_offt_*·*I_P_*	0.09275	0.04637	0.04645	1.00	0.364
*P_offt_*·*U_P_*	−0.09300	−0.04650	0.04645	−1.00	0.363
*I_P_*·*U_P_*	0.09275	0.04638	0.04645	1.00	0.364
-	*S*	*PRESS*	*R-Sq*	*R-Sq(pred)*	*R-Sq(adj)*
-	0.185800	1.76751	96.72%	66.45%	90.17%

**Table 6:** Estimated effects and coefficients for the number of peaks (coded units).

Term	Effect	Coef	SE Coef	*T*	*p*-Value
*Constant*	-	112.69	0.1008	1118.17	0.000
*P_ont_*	−22.62	−11.31	0.1008	−112.25	0.000
*P_offt_*	−22.62	−11.31	0.1008	−112.25	0.000
*I_p_*	−22.62	−11.31	0.1008	−112.25	0.000
*U_p_*	−22.62	−11.31	0.1008	−112.25	0.000
*P_ont_*·*P_offt_*	−0.12	−0.06	0.1008	−0.62	0.562
*P_ont_*·*I_P_*	−0.12	−0.06	0.1008	−0.62	0.562
*P_ont_*·*U_P_*	−0.12	−0.06	0.1008	−0.62	0.562
*P_offt_*·*I_P_*	−0.13	−0.06	0.1008	−0.62	0.562
*P_offt_*·*U_P_*	−0.13	−0.06	0.1008	−0.62	0.562
*I_P_*·*U_P_*	−0.12	−0.06	0.1008	−0.62	0.562
-	*S*	*PRESS*	*R-Sq*	*R-Sq(pred)*	*R-Sq(adj)*
-	0.403113	8.32	99.99%	99.90%	99.97%

**Table 7:** Regression analysis: roughness versus on, off, current and voltage.

Predictor	Coef	SE Coef	*T*	*p*
*Constant*	0.906	0.1500	6.03	0.000
*P_ont_*	0.0177	0.0037	4.77	0.001
*P_offt_*	0.0201	0.0029	6.91	0.000
*I_P_*	0.0300	0.0062	4.84	0.001
*U_P_*	0.0366	0.0053	6.90	0.000
-	*S* = 0.186	*R-Sq* = 92.8%	*R-Sq(adj)* = 90.2%	

**Table 8:** Regression analysis: number of peaks versus on, off, current and voltage.

Predictor	Coef	SE Coef	*T*	*p*
*Constant*	181.5	0.265	684.19	0.000
*P_ont_*	−0.9050	0.006571	−137.72	0.000
*P_offt_*	−0.7070	0.005134	−137.72	0.000
*I_P_*	−1.5083	0.01095	−137.72	0.000
*U_P_*	−1.2929	0.00939	−137.72	0.000
-	*S* = 0.3286	*R-Sq* = 100.0%	*R-Sq(adj)* = 100.0%	

**Table 9:** Analysis of variance for roughness (coded units).

Source	DF	Seq SS	Adj SS	Adj MS	*F*	*p*-Value
Main Effects	4	4.999	4.999	1.222	35.40	0.001
*P_ont_*	1	0.787	0.787	0.787	22.79	0.005
*P_offt_*	1	1.650	1.650	1.650	47.79	0.001
*I_P_*	1	0.810	0.810	0.810	23.46	0.005
*U_P_*	1	1.642	1.642	1.642	47.57	0.001
2-Way Interactions	6	0.207	0.207	0.035	1	0.510
*P_ont_·P_Offt_*	1	0.035	0.035	0.035	1	0.362
*P_ont_·I_P_*	1	0.035	0.035	0.035	1	0.363
*P_ont_·U_P_*	1	0.034	0.034	0.034	1	0.364
*P_offt_·I_P_*	1	0.034	0.034	0.034	1	0.364
*P_offt_·U_P_*	1	0.035	0.035	0.035	1	0.363
*I_P_·U_P_*	1	0.034	0.034	0.034	1	0.364
Residual Error	5	0.173	0.173	0.035		
Total	15					
Unusual Observations for Roughness						
Obs	StdOrder	Roughness	Fit	SE Fit	Residual	St Resid
15	6	1.874	2.106	0.154	−0.232	−2.24R

**Table 10:** Analysis of variance for the number of peaks (coded units).

Source	DF	Seq SS	Adj SS	Adj MS	*F*	*p*-Value
Main Effects	4	8190.25	8190.25	2047.56	12,600.38	0.000
*P_ont_*	1	2047.56	2047.56	2047.56	12,600.38	0.000
*P_offt_*	1	2047.56	2047.56	2047.56	12,600.38	0.000
*I_P_*	1	2047.56	2047.56	2047.56	12,600.38	0.000
*U_P_*	1	2047.56	2047.56	2047.56	12,600.38	0.000
2-Way Interactions	6	0.307	0.307	0.006	0.38	0.862
*P_ont_*·*P_offt_*	1	0.006	0.006	0.006	0.38	0.562
*P_ont_*·*I_P_*	1	0.006	0.006	0.006	0.38	0.562
*P_ont_*·*U_P_*	1	0.006	0.006	0.006	0.38	0.562
*P_offt_*·*I_P_*	1	0.006	0.006	0.006	0.38	0.562
*P_offt_*·*U_P_*	1	0.006	0.006	0.006	0.38	0.562
*I_P_*·*U_P_*	1	0.006	0.006	0.006	0.38	0.562
Residual Error	5	0.81	0.81	0.16		
Total	15	8191.44				
Unusual Observations for Roughness						
Obs	StdOrder	Roughness	Fit	SE Fit	Residual	St Resid
15	6	1.874	2.106	0.154	−0.232	−2.24R

**Table 11:** Estimated coefficients for roughness and for the number of peaks using data in uncoded units.

Term	Coef for Roughness	for Number of Peaks
Constant	1.121	181
*P_ont_*	0.0114	−0.881
*P_offt_*	0.0172	−0.689
*I_P_*	0.0182	−1.470
*U_P_*	0.0288	−1.263
*P_ont_·P_offt_*	0.00023	−3.12 × 10^−4^
*P_ont_·I_P_*	−0.000496	−6.70 × 10^−4^
*P_ont_·U_P_*	0.00042	−5.71 × 10^−4^
*P_offt_·I_P_*	0.00039	−5.21 × 10^−4^
*P_offt_·U_P_*	−0.00033	−4.46 × 10^−4^
*I_P_·U_P_*	0.00071	−0.00095

**Table 12:** Input and output electro-discharge texturing (EDT) parameters for target roughness *Ra_T,FR_*_1_ = 1.538 μm.

Parameters	Goal	Lower	Target	Upper	Weight	Import
Roughness	Target	1.538	1.539	3.714	1	1
**Starting Point**						
*P_ont_*	*P_offt_*	*I_p_*	*U_p_*			
4	6	4	7.5			
**Global Solution**				**Predicted Responses**		
*P_ont_*	*P_offt_*	*I_p_*	*U_p_*	Roughness *Ra* (µm)	Peak Count *Pc* (cm^−1^) (14)	Peak Count *Pc* (cm^−1^) (17)
4.29	6.32	4.47	8.1	1.539	159	156
Process capability indices *C_pk,UCL_*				2.77		
Process capability indices *C_pk,LCL_*					4.71	4.57

**Table 13:** Input and output EDT parameters for target roughness *Ra_T,FR_*_2_ = 2.5 μm.

Parameters	Goal	Lower	Target	Upper	Weight	Import
Roughness	Target	1.538	2.5	3.714	1	1
**Starting Point**				-		-
*P_ont_*	*P_offt_*	*I_p_*	*U_p_*	-	-	-
4	6	4	7.5	-	-	-
**Global Solution**				**Predicted Responses**		
*P_ont_*	*P_offt_*	*I_p_*	*U_p_*	Roughness *Ra* (µm)	Peak Count *Pc* (cm^−1^) (14)	Peak Count *Pc* (cm^−1^) (17)
4.86	6.97	15.1	25	2.5	121	118
Process capability indices *C_pk,UCL_*				2.78	-	-
Process capability indices *C_pk,LCL_*				-	6.78	6.44

**Table 14:** Input and output EDT parameters for target roughness *Ra_T,FR_*_3_ = 3.5 μm.

Parameters	Goal	Lower	Target	Upper	Weight	Import
Roughness	Target	1.538	3.5	3.714	1	1
**Starting Point**				-		-
*P_ont_*	*P_offt_*	*I_p_*	*U_p_*	-	-	-
4	6	4	7.5	-	-	-
**Global Solution**				**Predicted Responses**		
*P_ont_*	*P_offt_*	*I_p_*	*U_p_*	Roughness *Ra* (µm)	Peak Count *Pc* (cm^−1^) (14)	Peak Count *Pc* (cm^−1^) (17)
19.53	38	19	25	3.5	92	78
Process capability indices *C_pk,UCL_*				2.77	-	-
Process capability indices *C_pk,LCL_*				-	3.56	2.00
